# Emotions triggered by live arthropods shed light on spider phobia

**DOI:** 10.1038/s41598-021-01325-z

**Published:** 2021-11-15

**Authors:** Daniel Frynta, Markéta Janovcová, Iveta Štolhoferová, Šárka Peléšková, Barbora Vobrubová, Petra Frýdlová, Hana Skalíková, Petr Šípek, Eva Landová

**Affiliations:** 1 Department of Zoology, Faculty of Science, Charles University, Viničná 7, 128 43 Prague 2, Czech Republic; 2 National Institute of Mental Health, Topolová 748, 250 67 Klecany, Czech Republic

**Keywords:** Human behaviour, Psychology, Zoology

## Abstract

Spiders are mostly harmless, yet they often trigger high levels of both fear and disgust, and arachnophobia (the phobia of spiders) ranks among the most common specific animal phobias. To investigate this apparent paradox, we turned to the only close relatives of spiders that pose a real danger to humans: scorpions. We adopted a unique methodology in order to assess authentic emotions elicited by arthropods. Over 300 respondents were asked to rate live specimens of 62 arthropod species (including spiders, scorpions, cockroaches, and other insects) based on perceived fear, disgust, and beauty. We found that species’ scores on all three scales depended on the higher taxon as well as on body size. Spiders, scorpions, and other arachnids scored the highest in fear and disgust, while beetles and crabs scored the highest in beauty. Moreover, all chelicerates were perceived as one cohesive group, distinct from other arthropods, such as insects or crabs. Based on these results, we hypothesize that the fear of spiders might be triggered by a generalized fear of chelicerates, with scorpions being the original stimulus that signals danger.

## Introduction

For thousands of years, many animal species have evoked strong emotions such as fear, joy, disgust, or curiosity in humans^1,2^. From ancient times to this very day, countless depictions in myths, art, or popular literature testify to the deep interest humans have paid to certain animals. Among those, the most inconspicuous one is the spider. Spiders (Araneae) are often perceived negatively, evoking high levels of both fear and disgust in a wide variety of respondents^3–11^. Arachnophobia, the specific phobia of spiders, is one of the most common specific animal phobias, affecting 2.7–6.1% of the general population^12^. The exact aetiology of arachnophobia and spider-related fear is unknown, however, an evolutionary origin offers one of the potential explanations (^10^, but see also^13,14^).

Theoretical background to this idea was first formulated by Seligman^15^ in his hypothesis of biological preparedness (but see^16^). If spiders represented a real danger to our ancestors a fast fear response would be positively selected during the human evolution. Subsequently, this specific fear of spiders (or similar invertebrates) or at least a predisposition for fast associative learning of fear response^17^ would become genetically fixed through natural selection. Indeed, spiders and humans could share a long evolutionary history as spiders have been among the first terrestrial animals and are continuously inhabiting the Earth for at least 250 MY^18^. During this evolutionary history, their body plan has hardly changed^18^ and thus constituted a stable stimulus the human mind could adapt to. Later, more disgust-oriented hypothesis also offered an evolutionary explanation for negative emotions associated with spiders. Disease avoidance theory postulates that disgust originally evolved as a mechanism allowing to avoid consumption of harmful substances and further to avoid pathogens, parasites and other sources of contamination^19,20^. Under this paradigm, spiders would be seen as transmitters of diseases; an association that has cultural rather than biological origin^21^. For more detailed discussion on evolutionary origin of negative emotions associated with spiders see Landová et al.^10^.

Yet, Hauke and Herzig^22^ estimate that only 0.5% of all spider species are potentially dangerous to humans. Herman and Skokan^23^ state that only about 50 spider genera/species have chelicerae large enough to penetrate the human skin. Nentwig^24^ considers 42 of these spider taxa and concludes that bites of most of them cause only modest short-term effects. All these authors conclude that spiders are essentially harmless. Additionally, the coevolution of human ancestors and dangerous spider species is the key assumption in evolutionary oriented hypothesis of negative emotions associated with spiders. However, those few spider species that are dangerous to humans are usually not found in Africa (the place of human origin) but rather in Australia and Southern America (the continents inhabited by humans most recently). Some members of the genera *Latrodectus, Loxosceles* and *Hexophthalma* are found in Africa or the Middle East but the rest of the species from toxicologically important genera (9 genera) are absent in these regions^22,25^. Lastly, there is no evidence of spiders as disease transmitters. In light of these factors, the intense negative emotions commonly triggered by spiders seem rather odd.

To investigate the possible origin of the fear of spiders, we consider another chelicerate group—scorpions (Scorpiones). Scorpions share a similar body plan with spiders but unlike them, scorpions can pose a real threat to humans^26,27^. Every year, around 1.5 million scorpion bites are reported worldwide, with approx. 2600 of them being lethal^28^. North Africa and the Middle East are the most affected regions, but thousands of envenomations are registered all over the world except for Europe and Australia^28–31^. Scorpions are also an ancient, Palaeozoic, and morphologically conservative group distributed worldwide in warmer climates. While their venom probably originated as a predatory tool, an interesting new work^32^ shows venoms’ biochemical structure later evolved into such that acts specifically on mammalian ion channels. The beginning of this process coincides with the basal diversification of major scorpion mammal predators in the Cretaceous period^32^. The result of this evolutionary arms race makes some scorpion venoms especially effective (and painful) against mammals^32^. The prime example of scorpions possessing highly toxic and mammal-targeted venom is the family Buthidae (diversified about 130 MY ago). Thirteen out of fifteen toxicologically important scorpion genera belong to this family and eleven are native to Africa and the Middle East^31^ which makes the coevolution of scorpions and human ancestors possible. Interestingly, unlike spiders, scorpions also represent quite common model of mimicry (e.g., in lizards^33^ or phasmids^34^). In Batesian mimicry, the model is dangerous for the predator and signals its dangerousness in a way the mimic tries to replicate without the cost of being dangerous itself^35^. This strategy can only work if the predator recognizes the model as un unprofitable prey which supports the scorpion as a model of danger in the evolutionary history. Fear of scorpions therefore seems to be better warranted than fear of spiders and should be expected in the general population.

Nevertheless, fear of scorpions is very rarely explored in scientific literature and no specific phobia of scorpions is described. This paradox was previously considered by Vetter et al.^36^, who compared self-reported fear of spiders and scorpions in US university students using standardized questionnaires. They found that scorpion fear scores were equal to or significantly higher than spider fear scores. Similarly, in our previous study^10^, scorpion image evoked strong fear, comparable to images of robust spiders and tarantulas. Consequently, we hypothesize that the fear of spiders might be triggered by a more generalized fear of chelicerates, where scorpions are the original model which one should fear. However, no experimental study exploring this hypothesis has been conducted.

In this study, we collected live specimens of 62 arthropod species and asked almost 330 adult respondents to score them on fear, disgust, and beauty. Outside of a therapeutic context, psychological studies using live arthropods tend to focus on educational topics and use only a limited number of animal species (usually 3 to 5^7,8,37–39^). Therefore, this is by far the most comprehensive study on emotions triggered by live arthropods. While results obtained from questionnaires, images, videos, or virtual reality are of great value, the authentic experience with a live animal cannot be conveyed through any medium. Moreover, to the best of our knowledge, no study has compared emotions triggered by live arthropods to that triggered by their images.

In line with the focus of our study, about half of the used species were chelicerates—15 spiders, 10 scorpions, and 5 other arachnids. Two specific arthropod phobias have been described—the already mentioned arachnophobia and the phobia of cockroaches^40,41^. For this reason, we also included 10 species of cockroaches, and 10 species of other hemimetabolous insects (i.e., four phasmids, two locusts, two praying mantises, a true bug, and an earwig) as a control group. Several species of myriapods, crabs, and beetles were also added, to cover a wider range of arthropod morphotypes. In total, we used 62 invertebrate species (see “Methods” for details). All animals were scored on three scales: fear, disgust, and beauty. Fear is an emotion induced by perceived immediate danger or threat^42–44^, while disgust is an emotion expressed when rejecting something contagious, distasteful, or particularly unpleasant^19,20^. Fear and disgust were chosen as they are both specifically associated with spiders and commonly used in phobia research^3–11^. Beauty is an aesthetic value, a feature of an object that makes it pleasurable to perceive^45^. Because beauty is associated with pleasure, it can be viewed as a proxy for another of the basic emotions—joy^46,47^.

The aims of this study are as follows. (1) To test whether all chelicerates (i.e., spiders, scorpions, and other arachnids) are similarly salient stimuli. More specifically, we aim to test whether all chelicerate groups of stimuli score comparably high in fear and disgust, and whether they score the highest among all stimuli. Alternatively, scorpions might even exceed spiders and other arachnids in fear rating, as they are objectively the most dangerous of the stimuli. (2) To investigate the effect of body size on fear, disgust, and beauty scores of the stimuli, and to see whether this effect is universal across all animal groups or varies among the taxa. (3) To compare cockroaches with other hemimetabolous insects. Based on previous research^2,11^, we expect cockroaches to score the highest in disgust among hemimetabolous insects, approaching the disgust scores of spiders and possibly other chelicerates. (4) To explore whether some stimuli cluster together into cohesive groups and, if so, what are the relations between these clusters.

## Methods

### Selection of the stimuli

As the experimental stimuli, we used live specimens of 62 arthropod species, representing eight arthropod clades/groups: (1) spiders (15 spp.), (2) scorpions (10 spp.), (3) other arachnids (5 spp.), (4) cockroaches (10 spp.), (5) other hemimetabolous insects (10 spp.), (6) myriapods (6 spp.), (7) beetles (4 spp.), and (8) crabs (2 spp.). The species were selected as typical representatives of their respective groups, since we aimed to compare groups of stimuli (rather than individual stimuli) among each other. To achieve balanced design across the groups, we further considered the following factors: the size of the animal, its objective potential dangerousness, its rumoured dangerousness, and its area of origin. We avoided animals under 3 cm of body length, as they might have been difficult to observe for the respondents. We avoided larvae, primarily water species, and typically flying insects (e.g., bees, flies, butterflies, dragonflies). They were excluded because we find them difficult to compare to the typically non-flying insects and even more so to other flightless arthropods, including spiders and scorpions, our main objects of interest. For the full list of stimuli, see Supplementary Table S1 and Supplementary Fig. S1.

### Setting up the experiment

The experiment took place at the Faculty of Science, Charles University (17th–20th September 2020), and the National Institute of Mental Health (23rd–25th September 2020). The conditions in both testing sites were comparable. A day before the experiment, all live stimuli were weighted using an electronic scale (precision 0.001 g). The morning of the experiment, the stimuli were placed individually into identical plastic transparent boxes (19 × 13.5 × 6.5 cm) with breathing holes in the sidewalls and the floor covered by fine white sand. The lid was secured by transparent tape. As fast movement occurs mostly during exploration of a new environment, the boxes with stimuli were placed into the testing room a half an hour before the experiment to provide animals with sufficient time for habituation. All animals were provided with water. The boxes were numbered and placed in ascending order on tables arranged in a U shape, with approx. 20 cm spacing between adjacent boxes. A label with the scientific species name was placed next to each box. At the end of each day, animals were transferred back to their regular holding room and, if needed, they were provided with fresh water, food, or sand. We aimed to collect a complete scoring of at least 310 respondents in order to obtain enough cases (5 times more than variables) for subsequent multivariate analyses.

### Testing procedure

The testing procedure was completed by 329 respondents (186 women, 143 men, all adults aged 18–79, mean 35, median 32) of which 316 were Czechs and Slovaks and 13 were respondents from various European countries (foreign PhD students living in the Czech Republic for at least a year). The respondents were asked to evaluate each stimulus in terms of (1) fear (how fear-eliciting the stimulus is), (2) beauty (how beautiful the stimulus is), and (3) disgust (how disgust-eliciting the stimulus is). Each parameter was rated on a 7-point Likert scale (1—no fear/not at all beautiful/no disgust, 7—great fear/very beautiful/strong disgust^48^). Firstly, respondents were briefed about the nature of the experiment, and they gave a written consent with their personal data processing. Secondly, they were asked to fill in some personal information (name, gender, age, level of education, and field of education) and the experimental procedure was explained to them in detail. Afterwards, the respondents rated the stimuli; about half of the participants rated the stimuli in ascending order, the rest in reversed (descending) order. Lastly, respondents filled in two additional questionnaires: the 12-item Spider Phobia Questionnaire (SPQ-12; developed by Klorman et al.^49^; modified by Zsido et al.^50^), and the Disgust Scale-Revised (DS-R; developed by Haidt et al.^51^; modified by Olatunji et al.^52^; translated to Czech by Polák et al.^53^). The researchers were present and available for questions and assistance during the whole experiment. For Czech and Slovak respondents, the protocols were in Czech, for respondents of other nationalities, the protocols were in English.

### Data analysis

Firstly, we computed the two-way random, single score consistency intraclass correlations (standard ICCs) to quantify the amount of agreement between the respondents. Secondly, we computed the two-way random, average score consistency intraclass correlations (ICCs for averages^54,55^), which can be interpreted as estimates of the ‘accuracy’ of mean values. For basic characterization of the datasets, we compared the mean fear, disgust, and beauty scores of each stimulus using the post hoc Friedman-Neményi test. The results of these analyses confirmed that sample size (N = 329) and precision of estimated mean species ratings is sufficient for a subsequent linear modelling. We also calculated Pearson correlation coefficients between fear-disgust, fear-beauty, and disgust-beauty mean scores.

Next, we investigated the effect of body size and group identity on the mean rating using linear models (LM). In full models, the explanatory variables used were body size (expressed as natural logarithm of body weight), group identity (as defined in Supplementary Table S1), and their interaction. As the interaction did not prove significant (α = 0.05) in any of the models, it was removed. We also examined these relationships using generalized mixed effect models (GLMM) with binomial error distribution, where respondents’ identity was treated as a random factor. As LMs and GLMMs gave essentially the same results, we present only the results of the simpler LMs.

We performed exploratory factor analysis (FA) to find latent factors contributing to the rating of the examined stimuli. The FA was based on a correlation matrix and was done separately for each investigated scale (i.e., fear, disgust, and beauty). We used principal component extraction with Varimax normalized rotation. We determined the number of factors to retain by parallel analysis^56^. We then visualized the data’s structure using cluster analysis, likewise performed separately for each investigated scale. The distance matrix was calculated using Pearson correlations among species ratings, and tree diagrams were built using the Ward’s method. Lastly, we extracted gradients of variation constrained by respondents’ characteristics using the redundancy analysis (RDA^57^). The full models included the respondent’s gender, age, type of education, level of education, order of the stimuli, first rated emotion, SPQ-12 score, and the overall score of DS-R, as well as scores of its three subscales (Core, Contamination, and Animal Reminder disgust^52^).

The R programming language^58^ was used for computation of ICC (irr package^59^), Friedman-Neményi test (PMCMR package^60^), parallel analysis (EFA.dimensions package^56^), RDA analysis (vegan package^61^), and linear models. The software Statistica 9.1.^62^ was used to extract mean ratings of the stimuli, and to perform Pearson correlations, factor analyses, and cluster analyses. Full datasets associated with this study are available as Supplementary Information.

### Ethical note

All procedures performed in this study were carried out in accordance with the ethical standards of the appropriate institutional research committee (the Ethic Commission of National Institute of Mental Health, approval no. 175/20, granted on 16 September 2020). Informed consent was obtained from all participants included in the study. No additional animal ethics approval was required because experimental work was conducted with unregulated invertebrate species.

## Results

### Agreement among the respondents

Basic descriptive statistics for each species (mean, standard deviation) can be found in Supplementary Table S2.

We found sufficient agreement among the respondents. Standard ICCs quantifying agreement among individual respondents (N = 329) were 0.431 (CI_95%_: 0.353–0.528) for fear, 0.205 (CI_95%_: 0.156–0.277) for disgust, and 0.292 (CI_95%_: 0.229–0.380) for beauty.

ICCs for averages, reflecting the reliability of observed mean scores, were 0.996 (CI_95%_: 0.994–0.997), 0.988 (CI_95%_: 0.984–0.992), and 0.993 (CI_95%_: 0.990–0.995) for fear, disgust, and beauty data sets, respectively. Consequently, the majority of between-species comparisons of mean scores were significant (Friedman-Neményi post hoc test, see Supplementary Tables S3–5).

### Relationships between fear, disgust, and beauty

Rating of individual stimuli across all three scales was investigated using Pearson correlation. The mean fear and disgust scores of the stimuli were significantly correlated (r = 0.738, P < 0.0001), even after partialling out body weight (r_p_ = 0.688). The mean beauty scores were negatively correlated with mean disgust scores (r = − 0.438, P = 0.0004; r_p_ = − 0.602), but not with mean fear scores (r = 0.056, P = 0.6680; r_p_ = 0.070).

### Testing effects of body size and higher taxa

**Figure 1:**
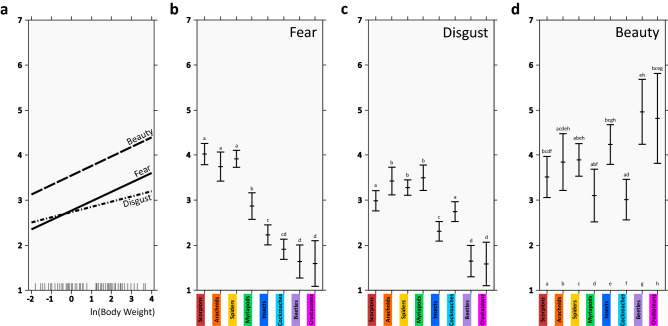
Effects of body size and higher taxa on mean fear, disgust, and beauty scores. (**a**) Effect of body size (expressed as natural logarithm of body weight) on mean fear, disgust, and beauty scores. The estimated slopes are 0.208 (fear), 0.115 (disgust), and 0.210 (beauty). The grey vertical lines on the x axis represent the cases. (**b**–**d**) Effect of higher taxa on mean fear (**b**), disgust (**c**), and beauty (**d**) scores after accounting for body size; estimated means and standard errors for each of the higher taxa are shown. In (**b**) and (**c**), letters above the whiskers signal no significant difference (α = 0.05) between the higher taxa sharing the same letter. In d, each higher taxon is assigned a unique letter; presence of the letter above the whiskers signals no significant difference (α = 0.05) between the respective taxa.

We employed linear models to assess the effects of body weight and higher taxon on mean scores of fear, disgust, and beauty (Fig. 1, Supplementary Table S6). There was no significant interaction between the examined factors in any of the full models, thus the final reduced models included only the main terms. The linear models confirmed the effect of body weight on fear (slope = 0.208, SE = 0.034, P <  < 0.0001), disgust (slope = 0.115, SE = 0.033, P <  < 0.0001) and beauty (slope = 0.210, SE = 0.067, P = 0.0084). The effect of higher taxon on all the scores was highly significant (all P <  < 0.0001).

In the model for fear scores, the examined taxa formed three distinct groups. Chelicerates scored the highest (spiders: 3.686, scorpions: 3.798, other arachnids: 3.523), followed by myriapods (2.643), and insects and crustaceans with the lowest scores (cockroaches: 1.685, other hemimetabolous insects: 2.005, beetles: 1.415, crabs: 1.361).

For disgust, the overall pattern was similar, but myriapods had the highest mean score (3.382), comparable to spiders (3.155) and other arachnids (3.300), while scorpions (2.873), cockroaches (2.631), and other hemimetabolous insects (2.185) were placed in intermediate positions. Similarly to fear scores, beetles and crabs had the lowest estimated disgust scores, 1.53 and 1.464, respectively.

Beauty estimated ratings followed a less clear pattern, with beetles (4.750), crabs (4.594), and other hemimetabolous insects (4.018) exhibiting the highest values, while myriapods (2.887) and cockroaches (2.802) occupied the lower part of the spectrum. Chelicerates received intermediate scores (3.678—spiders, 3.627—other arachnids, 3.295—scorpions).

**Figure 2:**
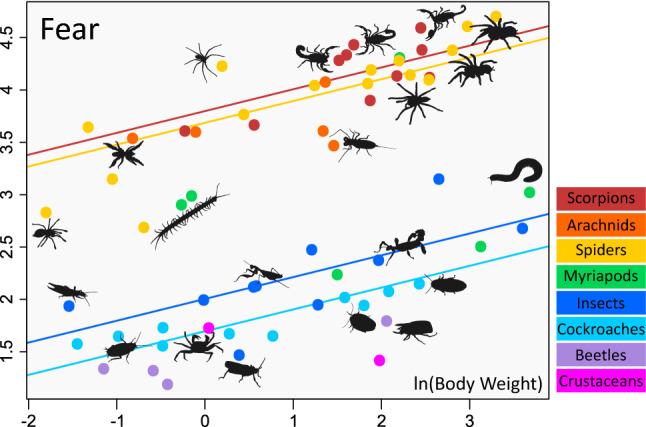
Relationship between body weight and observed mean fear scores. Each dot represents one stimulus (species), regression lines for the four focal groups are shown. The slope of the lines (0.208) does not differ between the groups.

The relationship between body weight and observed mean scores of stimuli is plotted in Fig. 2 (Fear) and Supplementary Figs. S2–3 (Disgust and Beauty).

### Exploratory analyses

**Figure 3:**
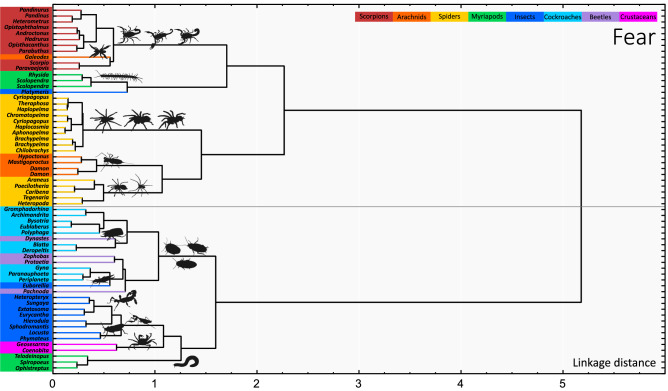
Cluster tree diagram based on fear ratings. All species are divided into two major clusters; the first one roughly corresponds to chelicerates (including spiders, scorpions, and other arachnids, but centipedes and the assassin bug also fall into this cluster) while the second one covers insects (beetles, cockroaches, and other hemimetabolous insects), crustaceans, and millipedes. Stimuli from all the predefined groups (represented by different colours) tend to cluster together suggesting participants responded consistently to the taxonomic groups. The only exception are myriapods which split into centipedes and millipedes.

In order to verify that the a priori defined taxa used as factors in the above models properly reflect the categories perceived by the respondents, we performed factor analysis. It confirmed that the major multivariate axes correspond well to the high-level taxa. Parallel analysis suggested extracting 5 factors for fear and disgust and 6 factors for beauty (Supplementary Table S7). Scores of spiders load highly on the first factor in each data set (i.e., fear, disgust and beauty). The other factors correspond to scorpions (all data sets), myriapods (all data sets, but millipedes contribute to another fear factor) and cockroaches (all data sets, but in the case of fear, other insects also contribute to this factor). Other hemimetabolous insects form a separate factor in beauty and disgust analyses, and another factor corresponds to beetles in beauty analysis. For explained variance and factor loadings see Supplementary Tables S8–10. To visualise multivariate relationships among the stimuli, we performed cluster analysis. It revealed a deep split between chelicerates and insects (+ crustaceans) as well as a strong tendency of the stimuli to cluster according to their higher taxa membership (scorpions, spiders + arachnids, myriapods, cockroaches, beetles, crustaceans, and the other hemimetabolous insects; see Fig. 3 for Fear, and Supplementary Figs. S4–5 for Disgust and Beauty).

We analysed the relationships between the respondents’ characteristics and their evaluation of the stimuli. RDA revealed that the contribution of constrained gradients was 0.145, 0.216, and 0.079 for the fear, disgust, and beauty data set, respectively. In all data sets, the most important factors were the SPQ-12 score and the Animal Reminder subscale of DS-R, both highly correlated with the first axis of variation. In the disgust dataset only, the Core subscale of DS-R also highly correlated with the first axis. The first axis explains almost all the variance in each data set. For full results of RDA see Supplementary Table S11. Notably, gender did not prove significant, although this was due to the SPQ-12 score (generally higher in women^63^) overshadowing the effect of gender. When SPQ-12 score was not included in the analysis, gender proved highly significant (results not shown).

## Discussion

The first objective of this study was to investigate the position of the chelicerate groups (i.e., spiders, scorpions, and other arachnids) on the fear, disgust, and beauty scales. We found that all chelicerates received the highest fear and disgust mean scores. This means they were perceived as the most salient stimuli among all arthropods, with the partial exception of myriapods, which scored comparably high on the disgust scale. Variation of species’ ratings on the beauty scale was greater than on the other two scales thus group identity might play a less important role in perceived beauty than in fear and disgust. In other words, species can be considered beautiful regardless of group membership and there is little to no difference between the mean beauty scores of the lowest and highest scoring groups. This being said, all chelicerate groups tended to score somewhere in the middle (see Fig. 1).

Secondly, we aimed to test the effect of animal’s body size on its rating. For all three scales, this effect was highly significant, with bigger animals receiving higher fear, disgust, and beauty scores. The effect was consistent across all higher taxa, albeit rather small (slope 0.115–0.21). This might be due the modest size difference between the smallest and the largest species. Nonetheless, the small range reflects the biological constraints on stimuli (respiratory systems of terrestrial arthropods are usually effective only up to a certain body size^64,65^), as well as respondents (people are able to view objects of at least a certain size). Therefore, we consider our estimated effects representative of terrestrial arthropod species. To sum it up, we found that the bigger the animal, the more intense the emotions it elicits, although its taxonomic identity affects the rating more (see Fig. 1). This result is generally consistent with the study of Zvarikova et al.^66^ where the experimentally enlarged body parts of spider stimuli elicited greater fear.

Thirdly, we investigated the position of cockroaches on the three scales. We found them scoring low in fear, intermediate in disgust, and very low in beauty, where they ranked as the least beautiful of all the arthropod groups. This was surprising to us, given that in the study of Grimaldos García et al.^41^, cockroaches were perceived very negatively even in comparison to spiders and snakes. However, that study did not look at fear or disgust, but rather valence and arousal ratings of a series of photographs. A wide diversity of snakes and spiders was used as stimuli, including colourful species or species with complex patterns, which usually contributes to positive evaluation of the animal^67–71^. Conversely, most cockroach stimuli were quite plain *Periplaneta* spp. (no. 38 in this study). This difference might have been the root cause of their very negative scores. To put it simply, cockroaches are generally perceived as rather ugly and therefore negatively, although they do not necessarily elicit strong negative emotions such as fear or disgust. In this light, our results are well compatible with theirs.

Fourthly, we used a series of multivariate exploratory analyses to uncover patterns in the datasets. In this regard, prime attention should be paid to the cluster analysis. Unlike simple modelling, this method groups stimuli based on the similarities in their ratings irrespective of whether a particular respondent rates them high or low. Its output, the tree diagrams, are a good graphical representation of the underlying data structure (see Fig. 3, Supplementary Figs. S4–5). Looking at the finest level of structure, terminal branches typically consist of species belonging to the same higher taxa, suggesting that people responded consistently to the taxonomic groups. It holds true for all the investigated scales but it is particularly pronounced in the fear and disgust datasets. This means that the selected stimuli are representative of their respective groups and we can meaningfully compare groups rather than individual stimuli, as we originally intended.

In the fear and disgust tree diagrams (Fig. 3, Supplementary Fig. S4), the deepest split is between the cluster of chelicerates and the cluster of insects plus crustaceans. This supports the notion that chelicerates are perceived as one cohesive group distinct from the other arthropods. In the introduction, we formulated a hypothesis that fear of spiders might be triggered by a more generalized fear of chelicerates, where scorpions are the original model which one should fear. In support of this hypothesis, we found that spiders, scorpions, and spider-like arachnids (whip spiders, whip scorpions, and camel spiders) are all rated as eliciting great fear and strong disgust. Moreover, their ratings tend to be very similar no matter how a particular respondent scored them, suggesting they are all recognized as members of one category.

However, a serious problem lies in the fact that we do not know what level of structure (if any particular one) is crucial for human perception of animals. One could argue that although spiders and scorpions cluster together, they still form their own separate subgroups making the hypothetical transfer of emotions originally targeted on only one of the groups less likely. Indeed, if spiders and scorpions did not form discrete units in the results of FA and cluster analysis, it would be a stronger support for our hypothesis. Other arachnids (whip spiders, whip scorpions and camel spiders) are interspersed in the chelicerate cluster, with some being closer to spiders and others to scorpions. This might lend more support to the notion that fear can be generalized based on superficial resemblance (similar results were found also in our previous work^10^).

Even if we accepted that the fear was generalised from one group to the other, we cannot decide what the direction of this generalization was. Nevertheless, only one path (i.e., from scorpions to spiders that is from the dangerous stimuli to the harmless one) make sense in the adaptive perspective. If fear of scorpions was generalized from fear of spiders, roots of the high fear of spiders would not be adaptive but rather a product of stochasticity, by-product of an unknown process or would not be a result of evolution per se. The last option would cover, for example, individual learning of fear through a bad experience or through witnessing another’s fear (vicarious learning^72^). We cannot reject this option because we did not ask respondents about their history. However, high fear response could both have adaptive evolutionary origin and be strengthened by individual and/or vicarious learning which is the case often seen in animals^73,74^. Although these arguments are not definitive, we still find that the presented results warrant further discussion about the possible shared origins of fear of spiders and scorpions (see also^36^).

Lastly, we should discuss why the specific phobia of scorpions has yet not been described in the literature concerned with mental health. One, a report bias can play an important role. The vast majority of reports estimating the abundance of specific animal phobias come from the western countries. However, scorpions are not very abundant in these regions and even in the areas where they are (e.g., SW of the USA), they are absent in larger cities. One could easily never meet a scorpion in the real life. Under such circumstances, high fear of scorpions could not manifest. Two, phobic patients themselves often consider their fear disproportionately high. Even though not all scorpions are dangerous, the dangerous species are not easily distinguishable from the harmless ones. Therefore, general public understandably believes the high fear of scorpions is justified. However, this reduces the chance it would be considered abnormal and/or a serious issue. Both these factors can contribute to the absence of reports on phobia of scorpions. Three, the specific phobias of different invertebrates are sometimes collectively labelled as “the animal phobia” or “the small animal phobia”^75,76^. This can unfortunately mask some interesting data regarding this topic.

Finally, we would like to point out one other interesting result. We found a strong positive correlation between fear and disgust ratings, which is very typical for many invertebrates^2,5,10,71,77,78^ but also found in some vertebrates (reptiles: 57%^79^, snakes: 38%^80^). If the selection favoured the fear of scorpions and simultaneously fear and disgust were principally correlated, we could formulate an alternative hypothesis as follows. Scorpions are predisposed to be an evolutionary relevant threat given their dangerousness and cohabitation with humans throughout their evolution. It might be that fear of scorpions is generalised onto spiders. As fear and disgust of animals are closely associated emotions (correlated together), it is possible that disgust too is transferred (generalised) from scorpion onto spiders. This is an alternative hypothesis to the one that disgust of spiders has been generalised from other parasites. So far, our results are inconclusive^2^, but our previous study^10^ shows that spiders are perceived as a group distinct from chelicerate parasites (a tick and a mite). Generalisation from parasites onto spiders would need to be very strong; this question needs to be addressed further in the future.

## Limitations and future directions

The study of animal phobias and emotions elicited by animals is a wide topic covering many fields, including psychology, psychiatry, neurobiology, or evolutionary biology. As a result, many interesting, related phenomena could not be discussed in detail in this study. For example, both fear and disgust can evoke avoidance behaviour. As scorpions elicit high levels of both, avoidance behaviour can be expected. The behavioural approach task (BAT) is especially suited for testing such behaviour and could represent an interesting new direction for future research. The biggest limitation of this study remains that, based on our results alone, we cannot decide whether fear of scorpions was generalized to fear of spiders or vice versa. Perhaps, this study might inspire other researchers interested in the enigmatic origins of arachnophobia. In our view, it is particularly interesting that scorpions, which present a more significant threat to humans from an evolutionary perspective, do not cause phobias (as opposed to snakes, for example), whereas spiders, which pose a negligible risk, are among the most common sources of animal phobias.

Throughout its evolutionary history, human mind had to face a huge diversity of animal species (and other potentially dangerous stimuli) and our ancestors successfully dealt with this task. Cognitive abilities as well as human emotions have helped us solve this uneasy task quickly and efficiently. This aspect (i.e., dealing with huge variability of animal stimuli via emotional reaction) which helps us decide whether we should avoid or approach the stimulus, has been neglected in the current psychological research. We believe that focus on natural variability of evolutionary relevant animal stimuli may bring new point of view for the future research focused on specific fears and phobias caused by animals.

## Conclusions

In this study, we focus on emotions—fear and disgust—and aesthetical preferences associated with eight groups of arthropods: scorpions, spiders, other arachnids, myriapods, cockroaches, other hemimetabolous insects, beetles, and crabs. With the aim to shed light on the origins of arachnophobia, we adopted a unique methodology, taking advantage of a wide variety of live arthropod stimuli in order to assess authentic emotions elicited by live animals. We hypothesize that the often (self-)reported strong fear of spiders (which otherwise seems inexplicable, as spiders are not dangerous) might be triggered by a much broader strong fear of chelicerates. As scorpions are the only truly dangerous chelicerate taxon, we suggest they might have been the original model humans were afraid of. In support of this hypothesis, we found that all chelicerates were perceived as one group, distinct from other arthropods, and also as very salient stimuli in both fear and disgust ratings. Therefore, the originally adaptive fear reaction to scorpions might have been extended to spiders and spider-like arachnids in human evolutionary history.

## Supplementary Information

Supplementary Information 1.

Supplementary Information 2.

## Data Availability

All data generated or analysed during this study are included in this published article in its Supplementary Information files.
